# K-ras/PI3K-Akt Signaling Is Essential for Zebrafish Hematopoiesis and Angiogenesis

**DOI:** 10.1371/journal.pone.0002850

**Published:** 2008-08-06

**Authors:** Lihui Liu, Shizhen Zhu, Zhiyuan Gong, Boon Chuan Low

**Affiliations:** 1 Cell Signaling and Developmental Biology Laboratory, Department of Biological Sciences, The National University of Singapore, Singapore, The Republic of Singapore; 2 Molecular Biology Laboratory, Department of Biological Sciences, The National University of Singapore, Singapore, The Republic of Singapore; RIKEN Genomic Sciences Center, Japan

## Abstract

The RAS small GTPases orchestrate multiple cellular processes. Studies on knock-out mice showed the essential and sufficient role of K-RAS, but not N-RAS and H-RAS in embryonic development. However, many physiological functions of K-RAS *in vivo* remain unclear. Using wild-type and fli1:GFP transgenic zebrafish, we showed that K-ras-knockdown resulted in specific hematopoietic and angiogenic defects, including the impaired expression of erythroid-specific gene *gata1* and *ße3-hemoglobin*, reduced blood circulation and disorganized blood vessels. Expression of either K-rasC40 that links to phosphoinositide 3-kinase (PI3K) activation, or Akt2 that acts downstream of PI3K, could rescue both hematopoietic and angiogenic defects in the K-ras knockdown. Consistently, the functional rescue by *k-ras* mRNA was significantly suppressed by wortmannin, a PI3K-specific inhibitor. Our results provide direct evidence that PI3K-Akt plays a crucial role in mediating K-ras signaling during hematopoiesis and angiogenesis *in vivo,* thus offering new targets and alternative vertebrate model for studying these processes and their related diseases.

## Introduction

*RAS* mutations are found in at least 20% of all human malignancies with *K-RAS* being the most frequently activated oncogene of all RAS proteins [1], [2]. Most of our understanding on the function and regulation of RAS stem from the over-expression of their constitutive active or dominant negative mutants or the other RAS signaling components. While useful, such approaches potentially lead to non-physiological effects [3]. Knock-out studies in mouse established that K-RAS is essential and sufficient for normal development while H-RAS and N-RAS are dispensable [4]–[7]. However, such studies could not conclusively identify the exact roles of RAS during normal tissue/organ development. Nonetheless, use of *K-ras* ^−/−^ fetal liver cells had shown that K-RAS signals to PI3K to regulate differentiation and proliferation of erythroid progenitor cells [8], [9]. Other evidence also point to a close functional association between wild type and oncogenic RAS, whereby wild type RAS could antagonize the function of oncogenic RAS [10]. All these call for a need to further address the physiological roles of RAS signaling at both the cellular and organismic levels.

The zebrafish *Danio rerio* is fast emerging as an excellent model for studying gene functions and signaling processes during development. Here we aim to define the physiological roles of wild-type K-ras in zebrafish through its specific gene knockdown, coupled with functional rescues by its pathway-specific mutant, their downstream effectors, and also the interference of rescue by pharmacological inhibitors.

## Results

### Zebrafish K-ras knockdown resulted in defective hematopoiesis and angiogenesis

**Figure 1:**
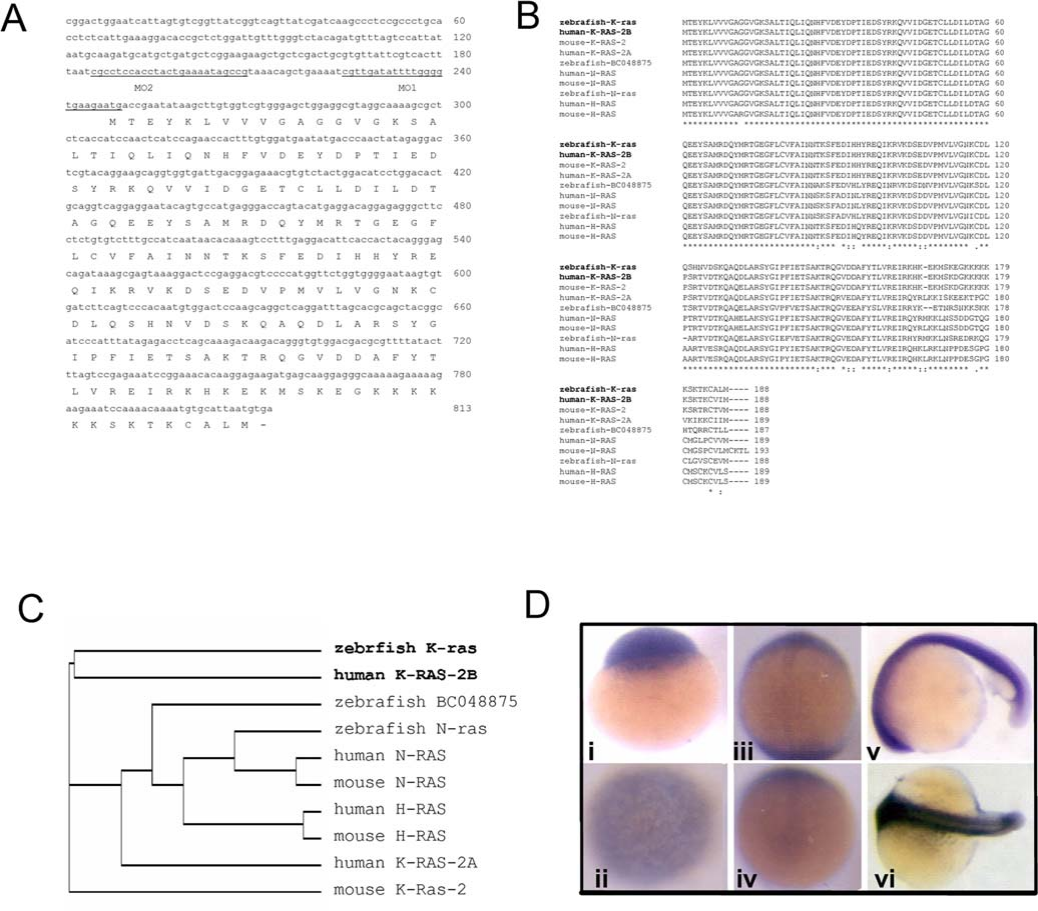
Sequence and expression analyses of zebrafish *k-ras*. (A) Zebrafish *k-ras* nucleotide and putative amino acid sequence. k-ras-MO1 and k-ras-MO2 binding sites are underlined. (B) Alignment of zebrafish K-ras with known Ras proteins of human, mouse and zebrafish. (C) Phylogenetic analysis of Ras proteins. (D) *k-ras* expression during embryonic development. *k-ras* transcripts were detectable from one cell stage (i) and then persist throughout the whole embryos (ii–vi). (i), one cell stage, side view; (ii), 3 hpf (hours-post fertilization), top view; (iii), 10 hpf, dorsal view, anterior to the top; (iv), 10 hpf, bottom view, dorsal to the top; (v), 20 hpf, lateral view, anterior to the left and dorsal to the top; (vi), 20 hpf, dorsal view, anterior to the left.

First, zebrafish *k-ras* cDNA (GenBank DQ486868) was isolated by reverse transcription-PCR. The encoded protein (Figure 1A) is highly homologous to human K-RAS2B and mouse K-RAS2, signified by a poly-lysine tract at its C-terminus (Figure 1A–1C). It is distinct from two other zebrafish Ras, N-ras [11] and BC048875 (Figure 1B and 1C), especially at their 5′UTR (supporting information Figure S1) that allows subsequent use of specific morpholino to knockdown K-ras. Zebrafish *k-ras* transcripts were detected from one-cell stage and continued to be detected throughout the whole embryos (Figure 1D). In adult, *k-ras* expression was detectable in most tissues (supporting information Figure S2).

**Figure 2:**
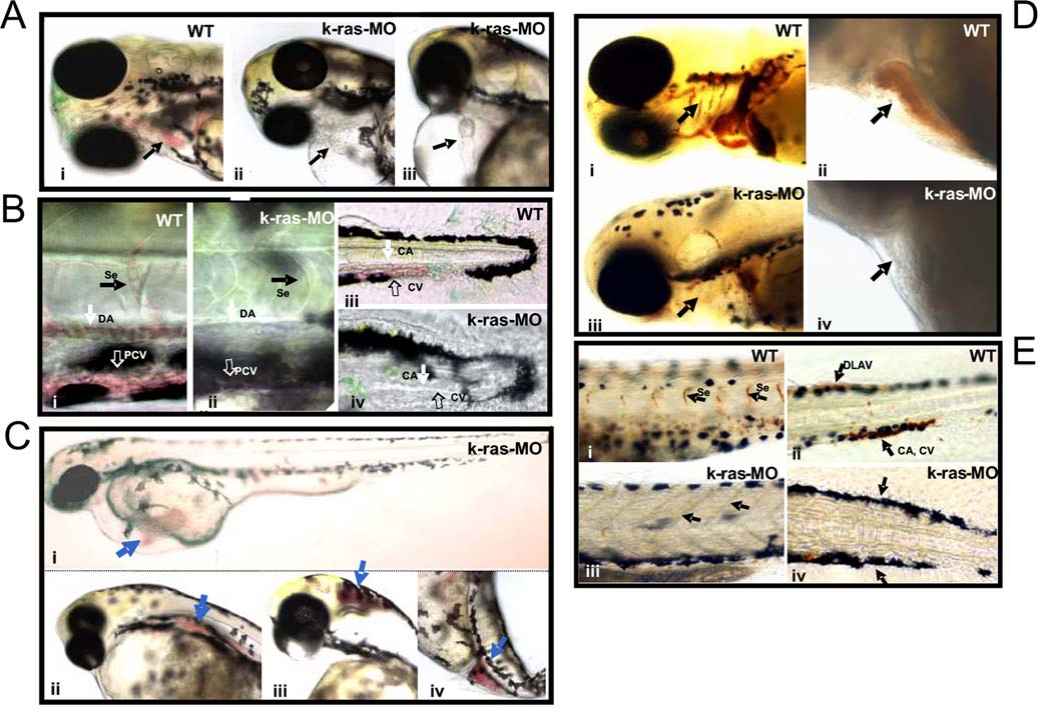
Disruption of zebrafish K-ras signaling resulted in the defective hematopoiesis. (A) k-ras-MO injected embryo showed empty heart, without or with few red blood cells inside (indicated by arrows in ii and iii), in comparison to wild type embryo, which showed plenty of red blood cells inside the heart (indicated by arrow in i). Embryos at 3 dpf (days-post fertilization). (B) Plenty of circulating red blood cells inside dorsal aorta (DA), posterior cardinal vein (PCV), inter-segmental vessels (Se), caudal artery (CA) and caudal vein (CV) in wild type embryos (i and iii), while no or less circulating red blood cells were found in k-ras-MO injected embryos inside DA, PCV, CA, CV and Se (ii and iv). Embryos at 3 dpf. (C) k-ras-MO injected embryos showed accumulated red blood cells in some sites that away from the circulation. Embryos at 3 dpf. (D) o-Dianisidine staining for wild type embryo showed hemoglobin positive cells inside branchial arches (indicated by arrow in i) and heart chambers (indicated by arrow in ii ), while k-ras-MO injected embryo showed less/negative o-Dianisidine staining for branchial arches (indicated by arrow in iii) and heart (indicated by arrow in iv). Embryos at 6 dpf. (E) o-Dianisidine staining for wild type embryo showed hemoglobin positive cells inside Se (indicated by arrows in i ), dorsal longitudinal anastomotic vessels (DLAV), CA and CV (indicated by arrows in ii), while k-ras-MO injected embryo failed to give the positive o-Dianisidine staining in these corresponding positions (indicated by arrows in iii and iv). Embryos at 6 dpf. All embryos shown are lateral view, with anterior to the left and dorsal to the top.

To identify the functional role of K-ras *in vivo*, translation of endogenous K-ras was suppressed by targeting *k-ras* mRNA with its specific antisense morpholino, k-ras-MO1 or k-ras-MO2 (Figure 1A). Optimal dose for microinjection was obtained that could result in specific defects but without gross lethality and global defects (materials and methods, Figure 2–[Fig pone-0002850-g003] Figure 4, and supporting information Table S1, Movie S1, S2, S3, S4, S5 and S6). From 24 hpf (hours-post fertilization) onwards, compared to control embryos, injected embryos showed reduced circulation of blood cells in the presence of a beating heart, albeit with lower beating rate (Figure 2A, Figure 2B, supporting information Figure S3 and Movie S1, S2, S3, S4, S5 and S6). Negligible or fewer circulating blood cells were seen inside the heart and blood vessels. This phenotype was observed in 76% of k-ras-MO1 injected embryos (75.8%±9.8, n>500, from 15 independent experiments). Moreover, accumulated red blood cells were often found at sites away from circulation (Figure 2C). As a negative control, one four-base mismatch morpholino k-ras-MO1-mis did not cause any of the above phenotypes as in k-ras-MO1 injected embryos (supporting information Figure S4). The specificity of K-ras knockdown was further confirmed by using a second morpholino, k-ras-MO2 that resulted in similar extents of defects in hematopoiesis (supporting information Table S1). Furthermore, the specificity and the efficiency of K-ras knockdown was confirmed by the reduced level of K-ras, rather than N-ras and H-ras, protein expression in K-ras MO injected embryos (supporting information Figure S5), and by the reduced level of the expression of a red fluorescent protein reporter fused downstream of *k-ras* 5′UTR in k-ras-MO injected embryos (supporting information Figure S6). Importantly, when *k-ras* mRNA was co-injected with k-ras-MO, such hematopoietic defects could be rescued effectively (Figure 5A and Figure 5B, supporting information Table S2), further supporting the specificity of K-ras knockdown. On the other hand, *k-ras* mRNA failed to rescue the gastrulation defects induced by the knock down of RhoA [12] (supporting information Figure S7), another small GTPase protein, demonstrating the specificity of the *k-ras* mRNA and the K-ras knock down.

**Figure 3 pone-0002850-g003:**
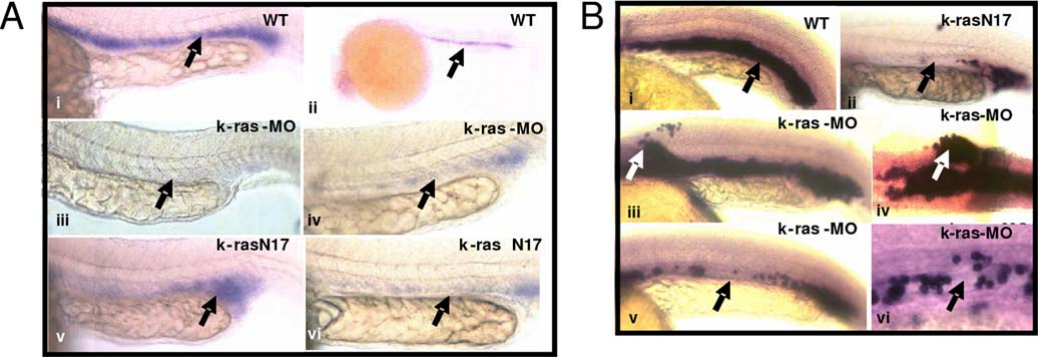
Disruption of zebrafish K-ras signaling resulted in the disruption of *gata-1* and *ße3-globin* expression. (A) K-ras knockdown or over-expression of K-ras-N17 resulted in the disruption of *gata-1* expression. Embryos at 20 hpf, lateral view, with anterior to the left and dorsal to the top. (B) K-ras knockdown or over-expression of K-ras-N17 resulted in the disruption of *ße3-globin* expression. Embryos at 24 hpf, lateral view, with anterior to the left and dorsal to the top.

**Figure 4:**
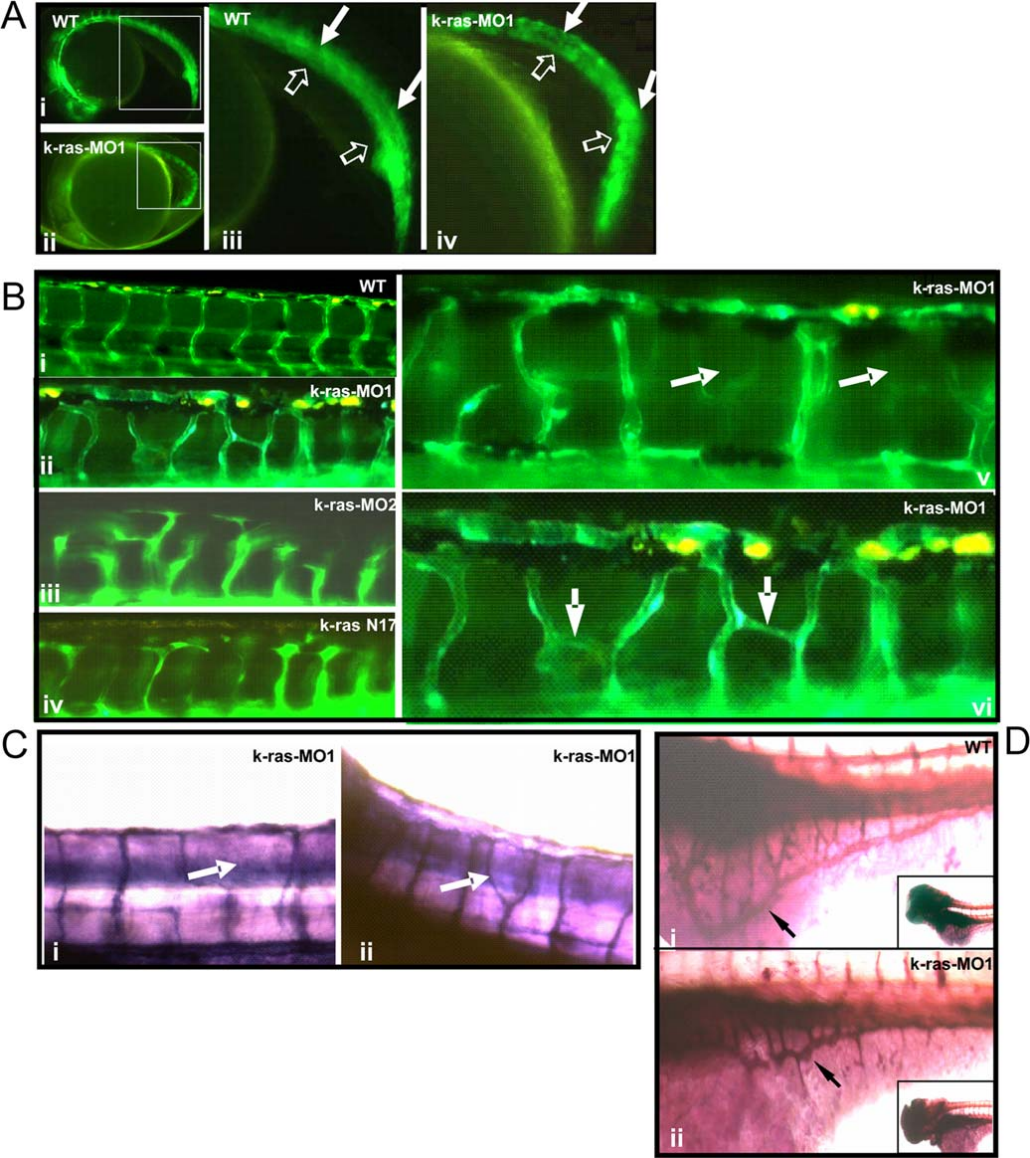
Disruption of zebrafish K-ras signaling resulted in the defective angiogenesis. (A) Both un-injected and k-ras-MO injected fli1-GFP embryos (22 hpf) showed normal development of dorsal aorta and caudal artery (indicted by solid arrows), posterior cardinal vein and caudal vein (indicated by empty arrows). Higher magnifications of the square area in (i) and (ii) were shown in (iii) and (iv) respectively. (B) Un-injected fli1-GFP embryo at 3 dpf showed well-organized inter-segmental vessels (i), while k-ras-MO1 injected (ii, v and vi), k-ras-MO2 injected (iii) or k-rasN17 injected (iv) embryos at 3 dpf showed aberrant and irregularly organized inter-segmental vessels. (C) Alkaline phosphatase staining for k-ras-MO1 injected embryos (3 dpf) showed aberrant trunk blood vessels. (D) Alkaline phosphatase staining showed well-organized SIV (sub-intestinal vein, indicated by arrow) in wild type embryo at 3 dpf (i), while disorganized SIV (indicated by arrow) in k-ras-MO1 injected embryo (ii). Inserted figures in i and ii showed the anterior part of the embryos. All embryos shown in lateral view, with anterior to the left and dorsal to the top.

**Figure 5:**
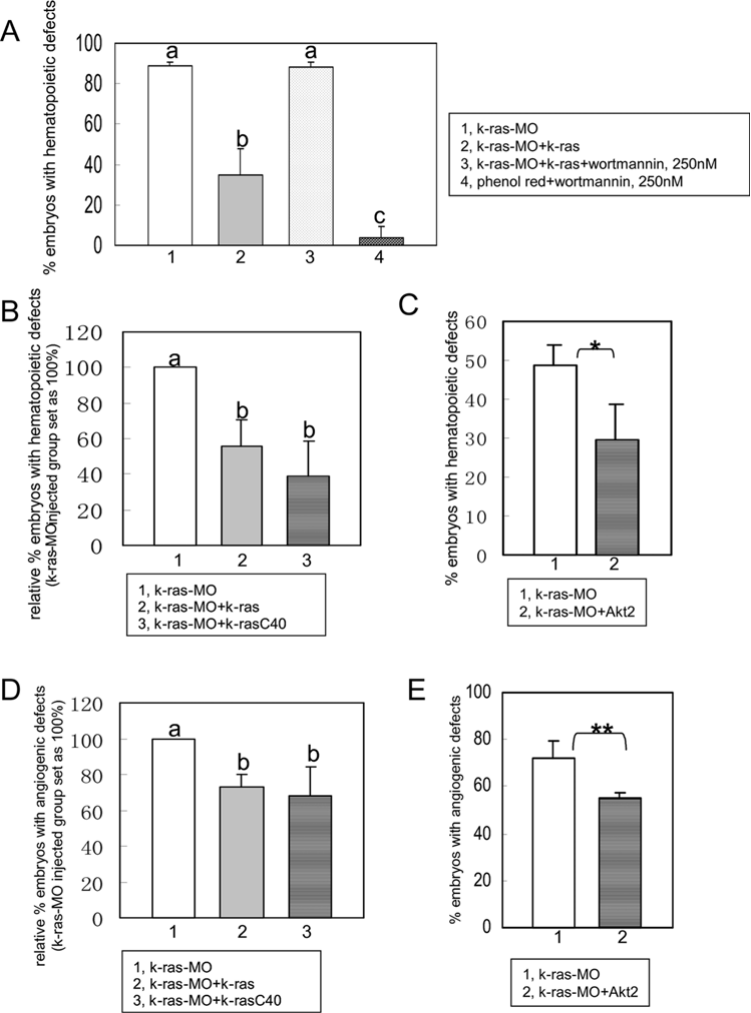
PI3K-Akt are crucial mediators for K-ras signaling in zebrafish hematopoiesis and angiogenesis. (A) K-ras knockdown could be rescued by *k-ras* mRNA, but this rescue was suppressed by wortmannin at lower dose (250 nM). At this concentration, wortmannin itself could not induce hematopoietic defects in controlled phenol-red injected group. Embryo numbers n1 = 37, n2 = 38, n3 = 43 and n4 = 46, from two independent sets of experiments. (B) Hematopoietic defects caused by K-ras knockdown could be rescued by wild type K-ras and K-ras mutant k-rasC40 respectively. Embryo numbers n1 = 475, n2 = 344 and n3 = 80, from >4 independent sets of experiments. (C) Hematopoietic defects caused by K-ras knockdown could be rescued by Akt2 effectively. Embryo numbers n1 = 156 and n2 = 129, from 4 independent sets of experiment. * indicates p<0.05. (D) Angiogenic defects caused by K-ras knockdown could be rescued by wild type K-ras and K-ras mutant k-rasC40 respectively. Embryo numbers n1 = 133, n2 = 92 and n3 = 62, from >2 independent sets of experiments. (E) Angiogenic defects caused by K-ras knockdown could be rescued by Akt2. Embryo numbers n1 = 36, n2 = 20, each group from 2 independent sets of experiments. ** indicates p<0.10. All data are means±SD (standard deviation). Values indicated by the same letter are not significantly different at p<0.01 for (A) and (B), and at p<0.05 for (D).

To further validate the hematopoietic defects, expression of erythroid-specific genes was examined. *In-situ* hybridization revealed the defects or partial loss of the expression of *gata1* (Figure 3), one well studied erythroid specific gene in early stage embryo. In addition, some ectopic *gata1* expression domains were observed as well in some embryos. Disrupted *gata1* expression was found in 58% (n = 42 in total n = 72) knock down embryos. In agreement with this, when we used *ße3-hemoglobin*, a gene normally expressed in differentiating erythrocytes within ICM (intermediate cell mass) from 15-somite stage, to evaluate the erythroid development, abnormal *ße3-hemoglobin* expression was noted in 43% (n = 13 in total n = 30) of the K-ras knock down embryos, including either the reduced expression of *ße3-hemoglobin* or some ectopic expression of *ße3-hemoglobin* (Figure 3). It hence indicates that the disruption of the expression pattern was not due to the failure of developing erythrocytes to express *gata1* or *ße3-hemoglobin*, but was due to the loss of developing erythrocytes in ICM and the misdistribution of the erythrocytes in some ectopic locations.

Consistently, lack of erythrocytes was further confirmed by o-Dianisidine staining, in agreement with the microscopic analysis for live embryos (Figure 2A and Figure 2B, supporting information Movie S1, S2, S3, S4, S5 and S6). Hemoglobinization appeared normal since all the existing red blood cells, including the circulating ones which were inside the blood vessels and those away from the circulation, showed the positive o-Dianisidine staining.

Since hematopoietic stem cells and angioblasts originate from bipotential precursors and thus the blood and blood vessel formation are closely connected in early stage [13], we set out to examine if the vascular-angiogenesis was also affected by K-ras knockdown. A vascular-specific transgenic line fli1:GFP, which allows immediate and direct *in-situ* monitoring of blood vessel formation [14] was used. k-ras-MO was injected into fli1:GFP embryos at 1–4 cell stage. GFP expression was not altered significantly until 22–24 hpf (Figure 4A). The major vessels, such as dorsal aorta, caudal artery, posterior cardinal vein and caudal vein, appeared normal, indicating normal vasculogenesis. But at later stages, angiogenesis started to be disrupted. By 48 hpf, defective vasculature was clearly visible. The regular normal trunk vessels (Figure 4B–i) were replaced by disorganized vascular channels (Figure 4B–ii to 4B–vi, Figure 4C). Particularly, sub-intestinal vein (SIV) was examined at 3 dpf (days-post fertilization), since the well-organized SIV at 72 hpf was regarded as one of the criteria to evaluate both angiogenic and anti-angiogenic effects [15] (Figure 4D). There, disorganized SIV, bearing obviously reduced size/numbers of vessel branches and/or ectopic blood vessels, accounted for 76% of k-ras-MO1 injected embryos (76.0%±10.2, n = 131, from 5 independent experiments) compared to only 17% basal defect level of wild type. Moreover, the same angiogenic defects could also be induced by k-ras-MO2 (Figure 4B–iii), demonstrating that the angiogenic defects are specifically resulted from K-ras knockdown. Furthermore, when *k-ras* mRNA was co-injected with k-ras-MO, numbers of embryo with disrupted SIV were reduced significantly (Figure 5).

To further demonstrate the essential role of normal Ras signaling, k-rasN17, one dominant negative mutant of K-ras, was expressed in zebrafish embryos. Consistently, it caused similar hematopoietic and angiogenic defects (Figure 3A–v, 3A–vi, 3B–ii, Figure 4B–iv and supporting information Table S1), strongly supporting that the defective hematopoiesis and angiogenesis result from the disruption of Ras signaling.

### PI3K-Akt is important in mediating K-ras signaling for both hematopoiesis and angiogenesis

Disruption of K-ras signaling caused defective hematopoiesis and angiogenesis during zebrafish embryonic development. Subsequently, to determine the involvements of the downstream effectors of Ras in these two processes, we analyzed the two major downstream pathways, PI3 Kinase pathway and MAP Kinase pathway respectively.

Firstly, zebrafish embryos were treated with PI3K inhibitor wortmannin. Wortmannin (1 µM) was able to cause defective blood and blood vessel formation, mimicking the defects in K-ras morphants (supporting information Figure S7, Table S3). This finding implies that signaling nodes at PI3K-Akt is important for zebrafish hematopoiesis and angiogenesis, and might be involved in mediating the downstream signals of K-ras. To prove it, we examined the ability of this inhibitor at sub-optimal levels to interfere with the functional rescue by *k-ras* mRNA for K-ras knockdown. Indeed, lower concentration of wortmannin (250 nM), which itself had no effect, could significantly nullify the rescue ability of *k-ras* mRNA (Figure 5A). This highlights the importance of PI3K for mediating K-ra*s* signaling during hematopoiesis. To substantiate this further, we checked the ability of functional rescue of K-ras mutant, k-rasC40, which is known to preferably activate PI3K; and Akt2, which acts downstream of PI3K [16], [17]. Consistently, k-rasC40 could significantly reduce the hematopoietic and angiogenic defects caused by K-ras knockdown (Figure 5B and 5D), and Akt2 also could reduce the extents of both hematopoietic and angiogenic defects in K-ras knockdown embryos (Figure 5C and 5E.). Taking together, all these results demonstrate that the normal signaling from K-ras to PI3K-Akt is essential for maintaining the normal processes during hematopoiesis and angiogenesis.

As a comparison, we are also analyzing another major downstream pathway of Ras, Mek-Erk1/2, which acts downstream of Raf [18]. The Mek inhibitor U0126 could not effectively block the *k-ras* mRNA rescue for K-ras knockdown at lower doses, although by itself at a higher dose (10 µM) it was able to induce the similar defects shown in K-ras morphants (data not shown and supporting information Figure S8, Table S3). Moreover, the analysis of the rescue ability of either Mek, or K-ras mutant K-rasS35, which specifically targets the effector Raf [16], [17], indicates a complexity of the involvement of Raf/Mek/Erk in regulating hematopoiesis and angiogenesis. For an example, in the rescue for hematopoietic defects, the rescue ability of K-rasS35 and Mek1 do not always follow the same trends (supporting information Figure S9), suggesting the possibility of other downstream target(s), rather than the Mek, being involved in mediating the Ras-Raf signaling. Furthermore, when constitutive active MEK1 (S218D/S222D) was introduced to the rescue, such mutant already led to global defects in the embryonic development (data not shown). On the other hand, when U0126 was used at its sub-optimal levels it did not significantly block the Akt2 rescue after the K-ras knock-down (data not shown), implying that the basal Mek/Erk activity may not be necessary for the Akt2 function in regulating these two processes. More Mek-specific knockdowns are now being developed in order to better understand the exact involvements of Mek/Erk either in isolation or in concert with Akt2.

**Figure 6:**
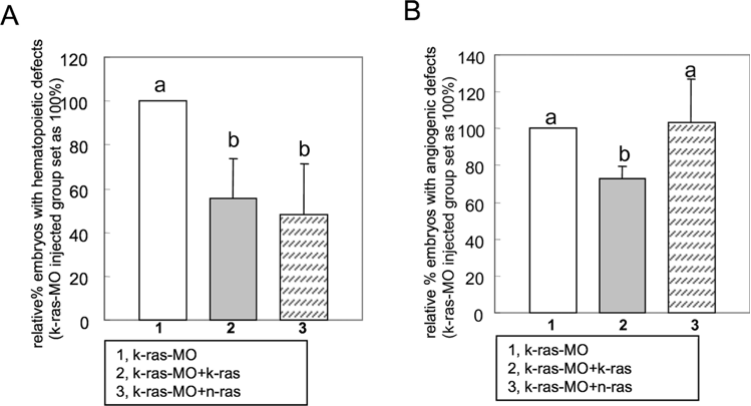
Zebrafish N-ras can rescue hematopoietic defects induced by K-ras knockdown, but not angiogenic defects. (A) Hematopoietic defects caused by K-ras knockdown could be rescued by zebrafish N-ras effectively. Embryo numbers n1 = 475, n2 = 344 and n3 = 131, from >3 independent sets of experiments. (B) Angiogenic defects caused by K-ras knockdown could not be rescued by zebrafish N-ras. Embryo numbers n1 = 133, n2 = 92 and n3 = 151, from 3 independent sets of experiments. All data are means±SD. Values indicated by the same letter are not significantly different at p<0.05.

To further elucidate the specificity versus redundancy in mediating Ras signaling in hematopoiesis and angiogenesis, zebrafish N-ras was introduced to rescue K-ras knockdown. The N-ras was shown to confer comparable functional rescue only for hematopoiesis but not for angiogenesis (Figure 6), thus, suggesting a unique function of K-ras which is not shared by N-ras. This is in agreement with the notion that distinct signal outputs from different RAS isoforms are necessary for diverse biological responses [19]. In essence, applying targeted gene knockdown for individual Ras isoforms in zebrafish will help clarify their distinct mechanisms, establish their relative contributions to normal physiological functions, and further analyze their potential downstream pathways. Such information will serve as the basis for developing the targeted therapeutics without disruption for other normal RAS signaling. Thus, zebrafish presents a significant model for faithfully reflecting the *in vivo* status of Ras signaling, bypassing the limitation by their over-expression that might re-route them to ectopic compartments.

## Discussion

Previous studies have revealed the roles of RAS in hematopoietic cell growth and differentiation, but the underlying signaling network *in vivo* is unclear. *K-ras* knockout mouse resulted in anemia and normal RAS signaling was required for erythroid differentiation, while K-RAS is the major regulator for AKT activation *in vitro* [4], [6], [8], [9]. Moreover, oncogenic RAS were known to associate with blood cancer and other blood disorders [20]–[22]. One hematopoietic transcription factor c/EBPß has been linked to RAS, which could be turned into an activator by RAS/MAPK signaling [23]. Erythropoietin (EPO) and Epo-receptor are also found to be able to activate RAS/RAF/MAPK and PI3K pathways [24]. Studies on human erythroid progenitors showed importance of PI3K for RAS, MEK and ERK activation, which were stimulated by EPO through a RAF-independent way [25]. All these suggest the extensive involvements of RAS signaling in hematopoiesis and highlight the importance and the complexity of PI3K-AKT and/or RAF/MEK/ERK in connecting to and mediating RAS signaling.

Our current studies first establish the *in vivo* function of PI3K-Akt as a mediator of K-ras signaling that regulates zebrafish hematopoiesis. This is consistent with the findings from *in vitro* studies in *K-ras* ^−/−^ fetal liver cells [9]. Alternatively, this may imply a potential cross-talk between PI3K and MAP kinase pathways, such as the studies in hematopoietic progenitor which demonstrated that MAP kinase pathway through RAS is PI3K-dependent and that PI3K drives RAF/MEK/ERK activation through RAF by a yet uncharacterized mechanism [26]. Likewise, it is possible that Mek/Erk may be under the control of other effectors, including PI3K, to exert its functional role. More evidence is however required to address this issue more conclusively.

RAS signaling has also been reported to link to angiogenesis, especially the tumor-angiogenesis. Oncogenic RAS through RAF/MEK/ERK and PI3K-AKT have been implicated in controlling VEGF expression [27]. Here, our results provide strong evidence that PI3K-Akt is an important common downstream regulator of K-ras signaling during both hematopoiesis and angiogenesis, lending further support on the interconnectivity of hematopoiesis and angiogenesis. Our results therefore supports the potential value of developing zebrafish as an ideal model for dissecting these two important biological processes mediated by K-ras signaling. In summary, our findings have established the important roles of K-ras signaling in zebrafish hematopoiesis and angiogenesis, with PI3K/Akt being an important mediator for these two processes. Within the complexity of Ras and GTPase signaling, it remains an important issue to determine whether other Ras effector pathways such as the Raf, Ral and others might be engaged separately or in concert with the PI3K/Akt. This and further comparative analyses for the involvement of other Ras isoforms and small GTPases, should help us better understand the signaling bases of the diseases *in vivo*, and to further develop the zebrafish as an alternative model for therapeutic screens.

## Materials and Methods

### Fish maintenance

Wild type and transgenic zebrafish were maintained by standard methods [28]. The transgenic line fli1-GFP was kind gift from Dr Ge Ruowen and was previously described [14]. All experiments on zebrafish were carried out at the National University of Singapore, in accordance with the National Advisory Committee for Laboratory Animal Research (NACLAR) Guidelines and in facilities licensed by the Agri-Food and Veterinary Authority of Singapore.

### Morpholinos

Antisense morpholino oligos (Gene-Tools) were designed to target the translational start (ATG) of *k-ras* as k-ras-MO1 and to target the upstream sequence of ATG in 5′UTR as k-ras-MO2 (Figure 1A).

### Microinjections

Morpholinos, mRNAs or plasmid constructs (supporting information for details) were injected into yolk of 1- to 4-cell stage zebrafish embryos. Doses for injection were titrated with lower toxicity and higher affectivity. Concentrations for injection are 600 µM for k-ras-MO1 and k-ras-MO1-mis, 1 mM for k-ras-MO2, 250 µM for RhoA-MO, 2.5 ng/µl of mRNA for k-ras, n-ras, k-rasC40 and k-rasS35, 18 ng/µl of mRNA for akt2, 40 ng/µl for Mek1 and 50 ng/µl for k-rasN17 respectively. Injection volume is around 2.3 nl.

### O-Dianisidine staining and alkaline phosphatase staining

Staining of hemoglobin by o-Dianisidine was carried out as described [29]. Sub-intestinal vein (SIV) development was evaluated by alkaline phosphatase staining as described [15].

### Inhibitor treatments

Wortmannin (Sigma) and U0126 (Promega) were dissolved in DMSO as stock and then diluted in egg water before use. Zebrafish embryos, wild type or injected ones, were treated with inhibitors from 3–4 hpf at the concentration indicated, controlled by DMSO treated wild type embryos, or by DMSO treated injected embryos. Embryos were maintained as standard method.

### Criteria for evaluating the status of zebrafish blood and blood vessel formation

For blood formation, we examined the circulation of live embryos at 30 hpf and 2 dpf, and then inspected the hemoglobin by o-Dianisidine staining at 2 dpf. Embryos bearing obvious reduction in circulation and o-Dianisidine staining in comparison to their wild type counterparts were regarded as abnormal. For blood vessel formation, we focused on checking SIV (sub-intestinal vein, indicated by arrows in Figure 4D) development by alkaline phosphatase staining at 3 dpf [15]. Embryos with disorganized SIV baskets, which lost the intact and bear obviously reduced numbers/size of vessel branches and/or had ectopic blood vessels, were regarded as abnormal.

### Data analysis

All data are presented as mean±SD (standard deviation). Statistic analysis was performed using one-way ANOVA. Differences were considered significant at p<0.01, P<0.05 or P<0.10 as indicated.

More methods were shown in Supporting Information **Text S1**.

## Supporting Information

Figure S1 Comparison of zebrafish k-ras, n-ras and BC048875.
(A) Comparison of amino acid sequences of three zebrafish Ras proteins, K-ras, N-ras and BC048875.
(B) Alignment of 5′UTRs of three zebrafish ras isoforms, k-ras, n-ras and BC048875. The morpholino targeting sites specific for k-ras, n-ras and BC048875 were highlighted with shade.
(10.00 MB TIF)

Figure S2 Expression analyses of zebrafish k-ras in tissues.
RT-PCR analysis of zebrafish k-ras, n-ras and BC048875 expression in adult zebrafish tissues. Most tissues examined, except spleen, show high or medium level of k-ras expression. Zebrafish n-ras and zebrafish BC048875 transcripts were also detectable in all tissues examined at variant levels.
(0.61 MB TIF)

Figure S3 Reduced heart beat rate was induced by K-ras knock-down, and it was able to be rescued by k-ras mRNA co-injection.
The observed heart beat rate (per 30 seconds) at 30 hpf (hours-post fertilization) of wild type embryos, k-ras-MO injected embryos, and k-ras-MO plus k-ras mRNA co-injected embryos respectively, showing the reduced heart beat rate caused by K-ras knock-down and the rescue by k-ras mRNA co-injection. Embryo numbers, n1 = 30, n2 = 26 and n3 = 38. Data are means±SD (standard deviation), *p<0.05.
Values indicated by the same letter are not significantly different at p<0.05.
(0.29 MB TIF)

Figure S4 The injection of mis-match k-ras morpholino (k-ras-MO-mis) could not induce the defects caused by k-ras morpholino.
(A) K-ras-MO-mis injected embryos showed significant difference from K-ras-MO injected embryos by the analysis of hematopoietic defects. Embryo numbers n1 = 106, n2 = 279 and n3>500 from >2 sets of independent experiments.
(B) K-ras-MO-mis injected embryos showed significant different from K-ras-MO injected embryos by the analysis of angiogenic defects. Embryo numbers n1 = 81, n2 = 117 and n3 = 113 from >2 sets of independent experiments.
Data are means±SD. Values indicated by the same letter are not significantly different at p<0.05.
(9.96 MB TIF)

Figure S5 Determination of K-ras, N-ras and H-ras protein level between wild type and k-ras-MO injected embryos.
K-ras-MO injected embryos (1 dpf, one day post fertilization) showed reduced K-ras protein expression compared to its wild type control, while the expression of N-ras and H-ras was not affected significantly, indicating the specificity and efficiency of K-ras knock-down.
(10.29 MB TIF)

Figure S6 RFP expression analysis at 20 hpf for k-ras-5′UTR-RFP injected embryos, indicating the targeting specificity of k-ras morpholino antisense oligo.
(A) Embryo injected with k-ras-5′UTR-RFP/PCS (red fluorescent protein reporter was down stream of K-ras 5′UTR and was cloned into PCS2 vector) construct, showing strong RFP signal.
(B) Embryo co-injected with k-ras-5′UTR-RFP/PCS and k-ras-MO1, showing very weak RFP signal, indicating the blockage of RFP protein expression by k-ras-MO1.
(C) Embryos from different treatments, showing the different RFP strength under the same exposure. These embryos were (i), injected with k-ras-5′UTR-RFP/PCS alone; (ii), co-injected with k-ras-5′UTR-RFP/PCS and k-ras-MO1; and (iii), wild type embryo with no injection.
(4.05 MB TIF)

Figure S7 k-ras mRNA could not rescue the gastrulation defects induced by RhoA knock down.
RhoA-MO injection can induce gastrulation defects [12] and these defects could not be rescued by the co-injection of k-ras mRNA. Embryos were observed at 1-somite stage. Embryo numbers n1 = 113, n2 = 112, from 2 sets of independent experiments. Data are means±SD. Values indicated by the same letter are not significantly different at p<0.05.
(9.69 MB TIF)

Figure S8. PI3K inhibitor wortmannin or MEK inhibitor U0126 could induce hematopoietic and angiogenic defects similar to the defects induced by K-ras knock-down.
(A) Either wortmannin or U0126 treatment were able to cause the hematopoietic defects. These defects include empty heart, with no or few red blood cells inside heart (indicated by arrows in ii and iii, compared to wild type in i), reduced or lack of normal circulation and reduced number of circulating red blood cells (indicated by arrows in v and vi, compared to wild type in iv), and accumulation of blood cells in some sites away from the circulation (indicated by arrows in vii and viii). All embryos were observed at 4 dpf (days-post fertilization), lateral view, anterior to the left and dorsal to the top.
(B) o-Dianisidine staining for wortmannin or U0126 treated embryos, showing loss or reduction of hemoglobin positive cells overall, especially inside heart and in yolk sac (indicated by empty arrows and block arrows respectively in ii, iii, v and vi, compared to wild type embryos in i and iv). Except for grouped embryos, all other embryos are lateral view, anterior to the left and dorsal to the top. Embryos were observed at 6 dpf.
(C) Either wortmannin or U0126 treatment were able to cause angiogenic defects. Inhibitor treatment for fli1-GFP embryos resulted in disorganized blood vessels, including the missing segmental vessels and/or bearing ectopic vessel sprouts (indicated by arrows in i and ii), similar to the defects caused by K-ras knock-down. Embryos were observed at 4 dpf, lateral view, anterior to the left and dorsal to the top.
(5.06 MB TIF)

Figure S9 Raf is involved in mediating K-ras signaling for both hematopoiesis and angiogenesis, while Mek might be only involved in angiogenesis, but not in hematopoiesis.
(A) Hematopoietic defects caused by K-ras knockdown could be rescued by wild type k-ras and k-ras mutant k-rasS35 respectively, but not Mek1. Embryo numbers n1>500, n2 = 475, n3 = 142 and n4 = 204, from >2 independent sets of experiments.
(B) Angiogenic defects caused by K-ras knockdown could be rescued by wild type k-ras, k-ras mutant k-rasS35 and Mek1 respectively. Embryo numbers n1 = 133, n2 = 92, n3 = 39 and n4 = 89 from >2 independent sets of experiments.
All data are means±SD (standard deviation). Values indicated by the same letter are not significantly different at p<0.05.
(7.49 MB TIF)

Movie S1 Beating heart filled with plenty of red blood cells in wild type embryo. Embryo at 2 dpf (days-post fertilization).
(6.14 MB MPG)

Movie S2 Beating heart filled with few red blood cells in k-ras-MO injected embryo. Embryo at 2 dpf.
(6.00 MB MPG)

Movie S3 Plenty of red blood cells circulating inside dorsal aorta and posterior cardinal vein in wild type embryo. Embryo at 2 dpf.
(6.41 MB MPG)

Movie S4 Few red blood cells circulating inside dorsal aorta and posterior cardinal vein in k-ras-MO injected embryo. Embryo at 2 dpf.
(7.98 MB MPG)

Movie S5 Plenty of red blood cells circulating inside caudal artery and caudal vein in wild type embryo. Embryo at 2 dpf.
(5.75 MB MPG)

Movie S6 Few red blood cells circulating inside caudal artery and caudal vein in k-ras-MO injected embryo. Embryo at 2 dpf.
(5.75 MB MPG)

Text S1 (0.05 MB DOC)

Table S1 Statistic results summarizing similar hematopoietic defects induced by k-ras-MO1, k-ras-MO2 and k-ras-N17 respectively, indicating that the hematopoietic defects are closely related to the disruption of K-ras signaling.
(0.03 MB DOC)

Table S2 Hematopoietic defects induced by K-ras knock-down could be partially rescued by over-expression of k-ras mRNA, suggesting the specificity of K-ras knock-down.
(0.03 MB DOC)

Table S3 Treatments with wortmannin or U0126 for zebrafish embryos could induce the hematopoietic defects and angiogenic defects, which phenocopy the functional loss of K-ras.
(0.03 MB DOC)
