# Supplementary figures and images for: K-ras/PI3K-Akt Signaling Is Essential for Zebrafish Hematopoiesis and Angiogenesis
Source: PLoS One. 2008 Aug 6;3(8):e2850. doi: 10.1371/journal.pone.0002850 (PMC2483249; doi:10.1371/journal.pone.0002850)

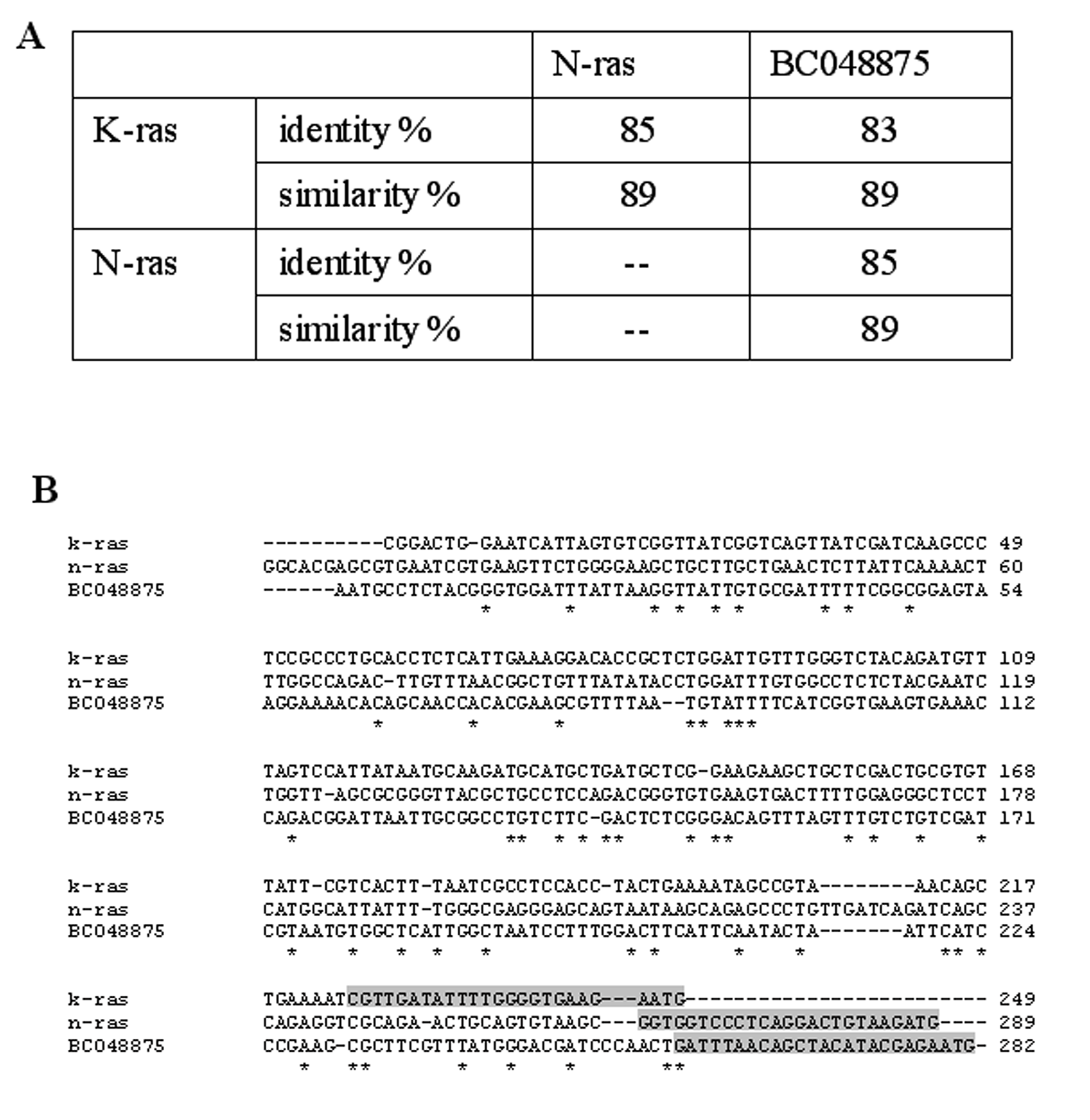

Supplement: Figure S1 — Comparison of zebrafish k-ras, n-ras and BC048875. (A) Comparison of amino acid sequences of three zebrafish Ras proteins, K-ras, N-ras and BC048875. (B) Alignment of 5′UTRs of three zebrafish ras isoforms, k-ras, n-ras and BC048875. The morpholino targeting sites specific for k-ras, n-ras and BC048875 were highlighted with shade. (10.00 MB TIF) [file pone.0002850.s001.tif]

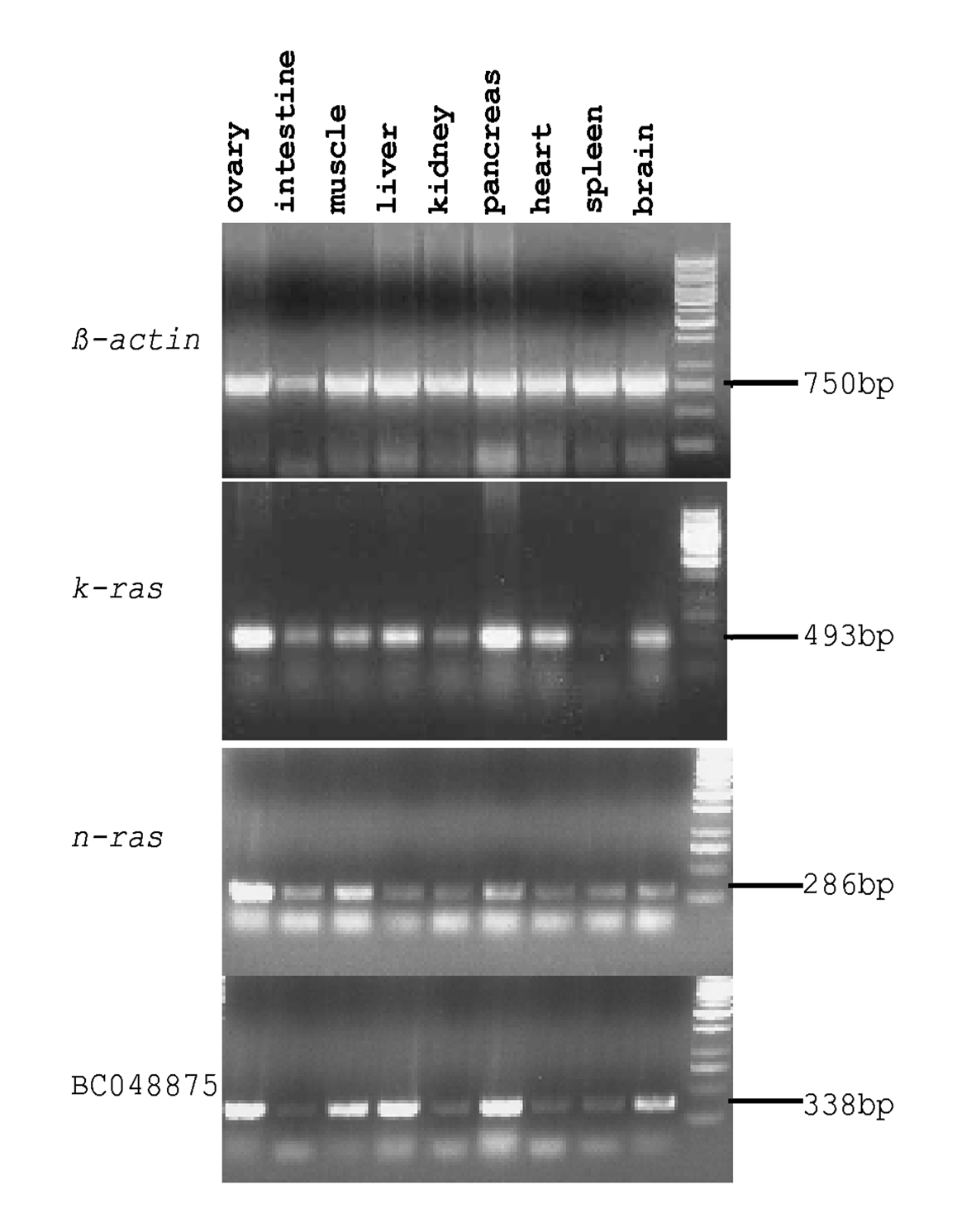

Supplement: Figure S2 — Expression analyses of zebrafish k-ras in tissues. RT-PCR analysis of zebrafish k-ras, n-ras and BC048875 expression in adult zebrafish tissues. Most tissues examined, except spleen, show high or medium level of k-ras expression. Zebrafish n-ras and zebrafish BC048875 transcripts were also detectable in all tissues examined at variant levels. (0.61 MB TIF) [file pone.0002850.s002.tif]

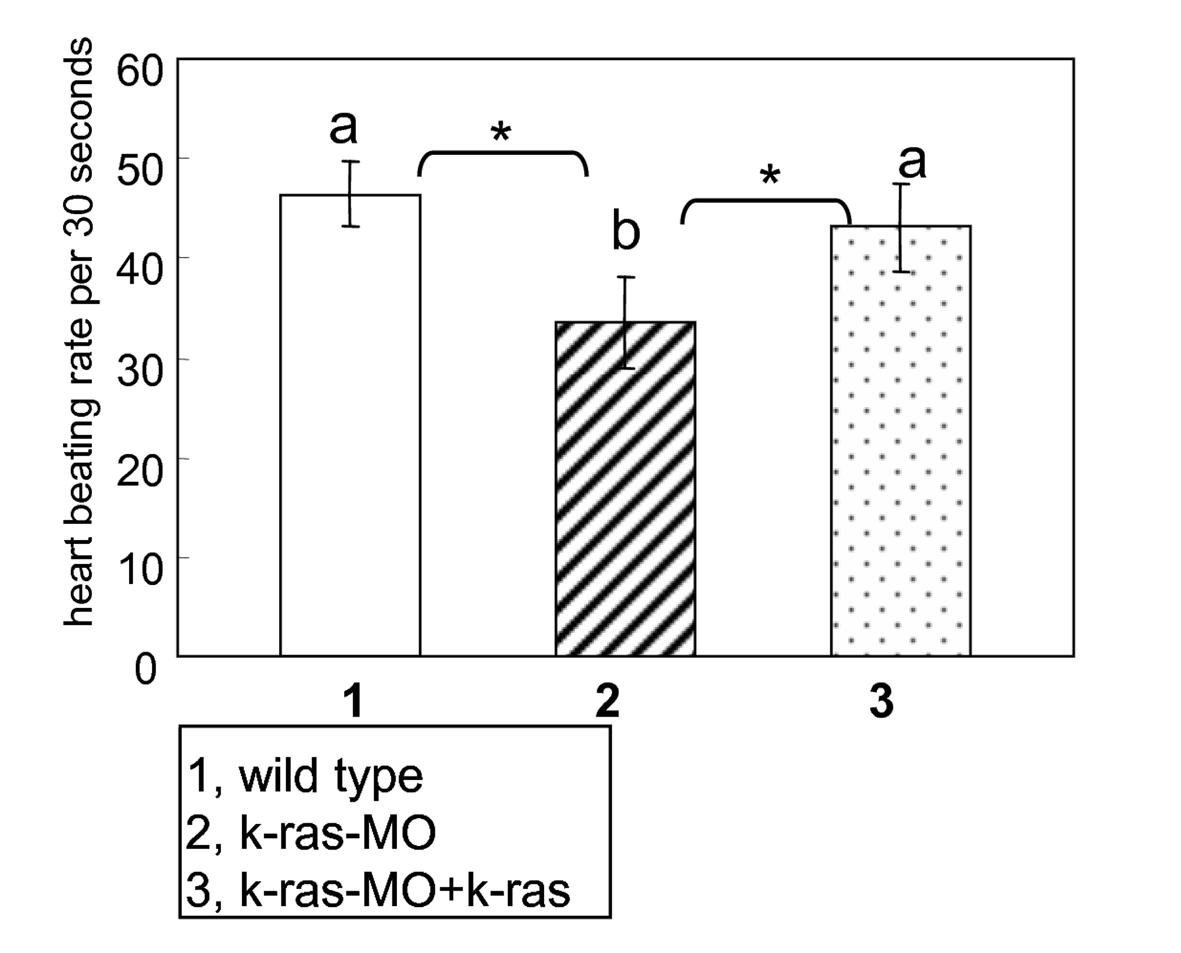

Supplement: Figure S3 — Reduced heart beat rate was induced by K-ras knock-down, and it was able to be rescued by k-ras mRNA co-injection. The observed heart beat rate (per 30 seconds) at 30 hpf (hours-post fertilization) of wild type embryos, k-ras-MO injected embryos, and k-ras-MO plus k-ras mRNA co-injected embryos respectively, showing the reduced heart beat rate caused by K-ras knock-down and the rescue by k-ras mRNA co-injection. Embryo numbers, n1 = 30, n2 = 26 and n3 = 38. Data are means±SD (standard deviation), *p<0.05. Values indicated by the same letter are not significantly different at p<0.05. (0.29 MB TIF) [file pone.0002850.s003.tif]

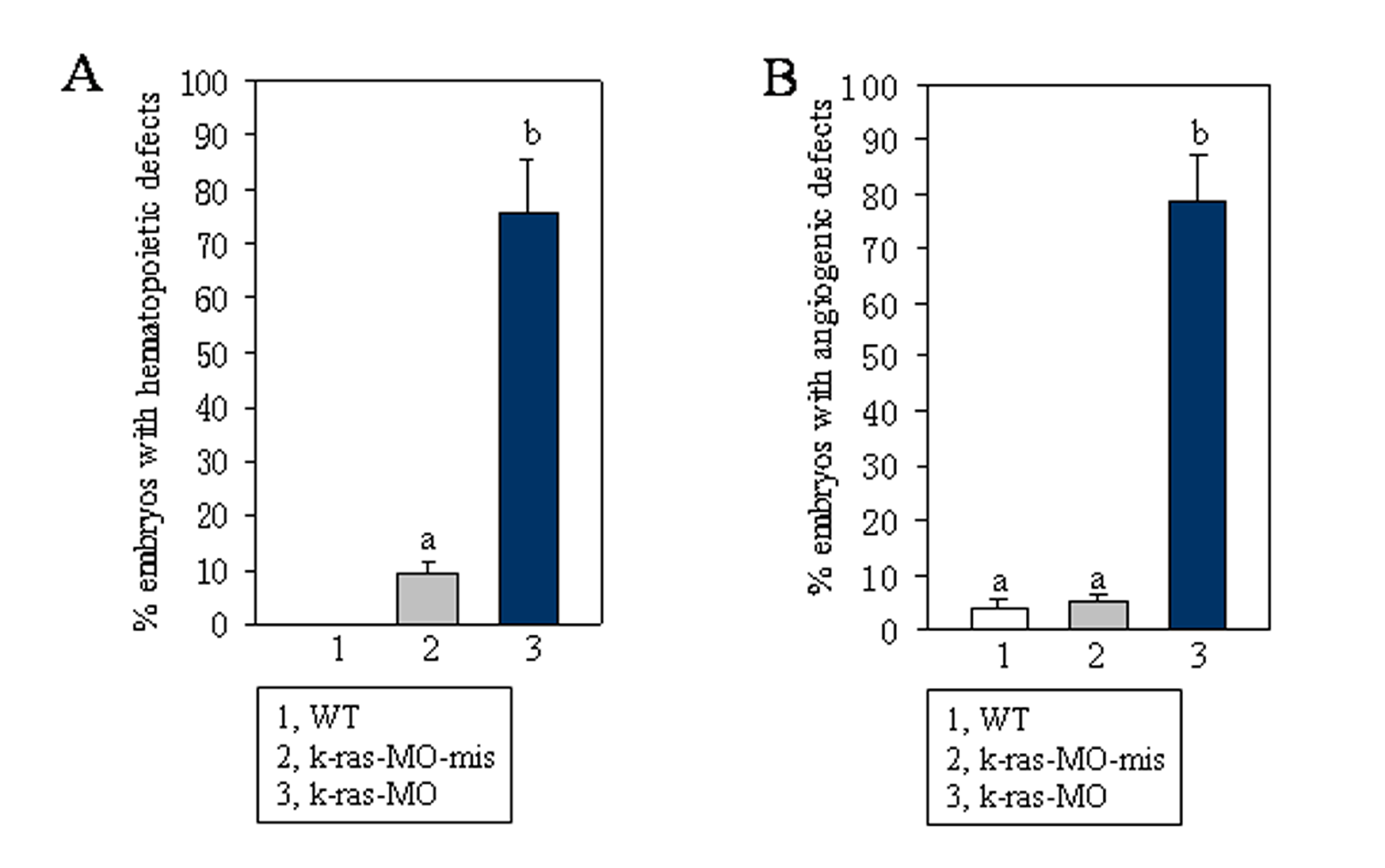

Supplement: Figure S4 — The injection of mis-match k-ras morpholino (k-ras-MO-mis) could not induce the defects caused by k-ras morpholino. (A) K-ras-MO-mis injected embryos showed significant difference from K-ras-MO injected embryos by the analysis of hematopoietic defects. Embryo numbers n1 = 106, n2 = 279 and n3>500 from >2 sets of independent experiments. (B) K-ras-MO-mis injected embryos showed significant different from K-ras-MO injected embryos by the analysis of angiogenic defects. Embryo numbers n1 = 81, n2 = 117 and n3 = 113 from >2 sets of independent experiments. Data are means±SD. Values indicated by the same letter are not significantly different at p<0.05. (9.96 MB TIF) [file pone.0002850.s004.tif]

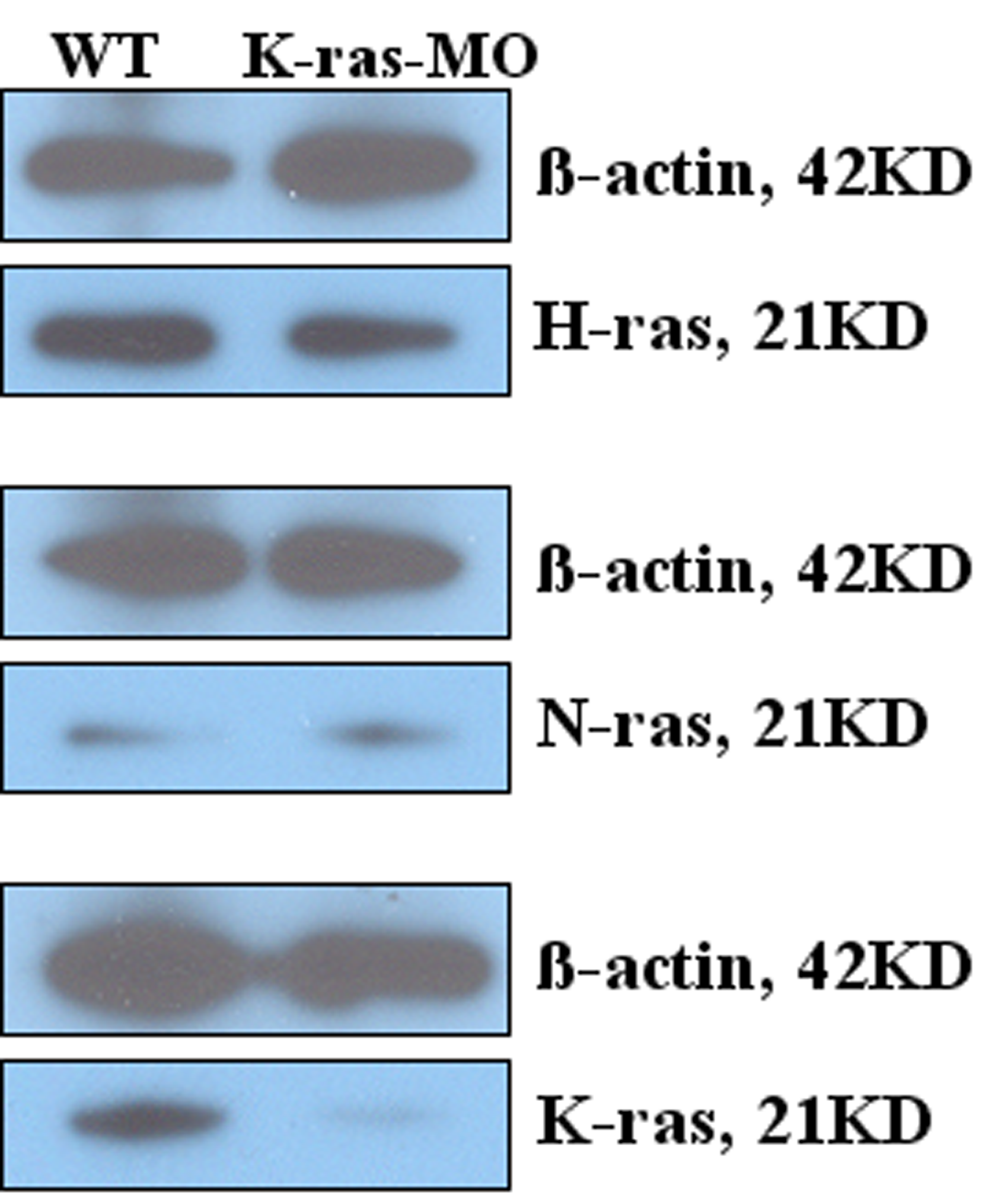

Supplement: Figure S5 — Determination of K-ras, N-ras and H-ras protein level between wild type and k-ras-MO injected embryos. K-ras-MO injected embryos (1 dpf, one day post fertilization) showed reduced K-ras protein expression compared to its wild type control, while the expression of N-ras and H-ras was not affected significantly, indicating the specificity and efficiency of K-ras knock-down. (10.29 MB TIF) [file pone.0002850.s005.tif]

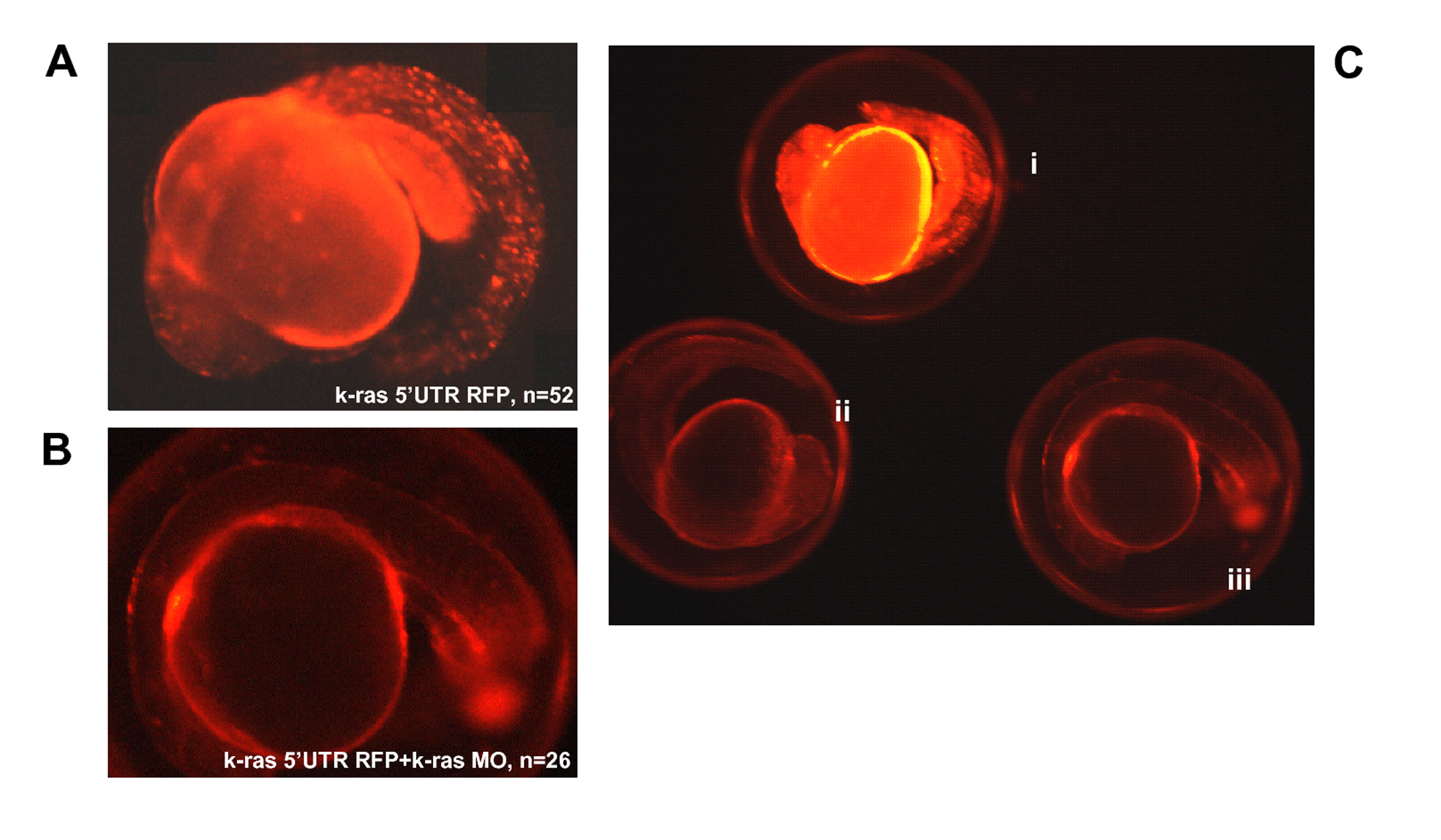

Supplement: Figure S6 — RFP expression analysis at 20 hpf for k-ras-5′UTR-RFP injected embryos, indicating the targeting specificity of k-ras morpholino antisense oligo. (A) Embryo injected with k-ras-5′UTR-RFP/PCS (red fluorescent protein reporter was down stream of K-ras 5′UTR and was cloned into PCS2 vector) construct, showing strong RFP signal. (B) Embryo co-injected with k-ras-5′UTR-RFP/PCS and k-ras-MO1, showing very weak RFP signal, indicating the blockage of RFP protein expression by k-ras-MO1. (C) Embryos from different treatments, showing the different RFP strength under the same exposure. These embryos were (i), injected with k-ras-5′UTR-RFP/PCS alone; (ii), co-injected with k-ras-5′UTR-RFP/PCS and k-ras-MO1; and (iii), wild type embryo with no injection. (4.05 MB TIF) [file pone.0002850.s006.tif]

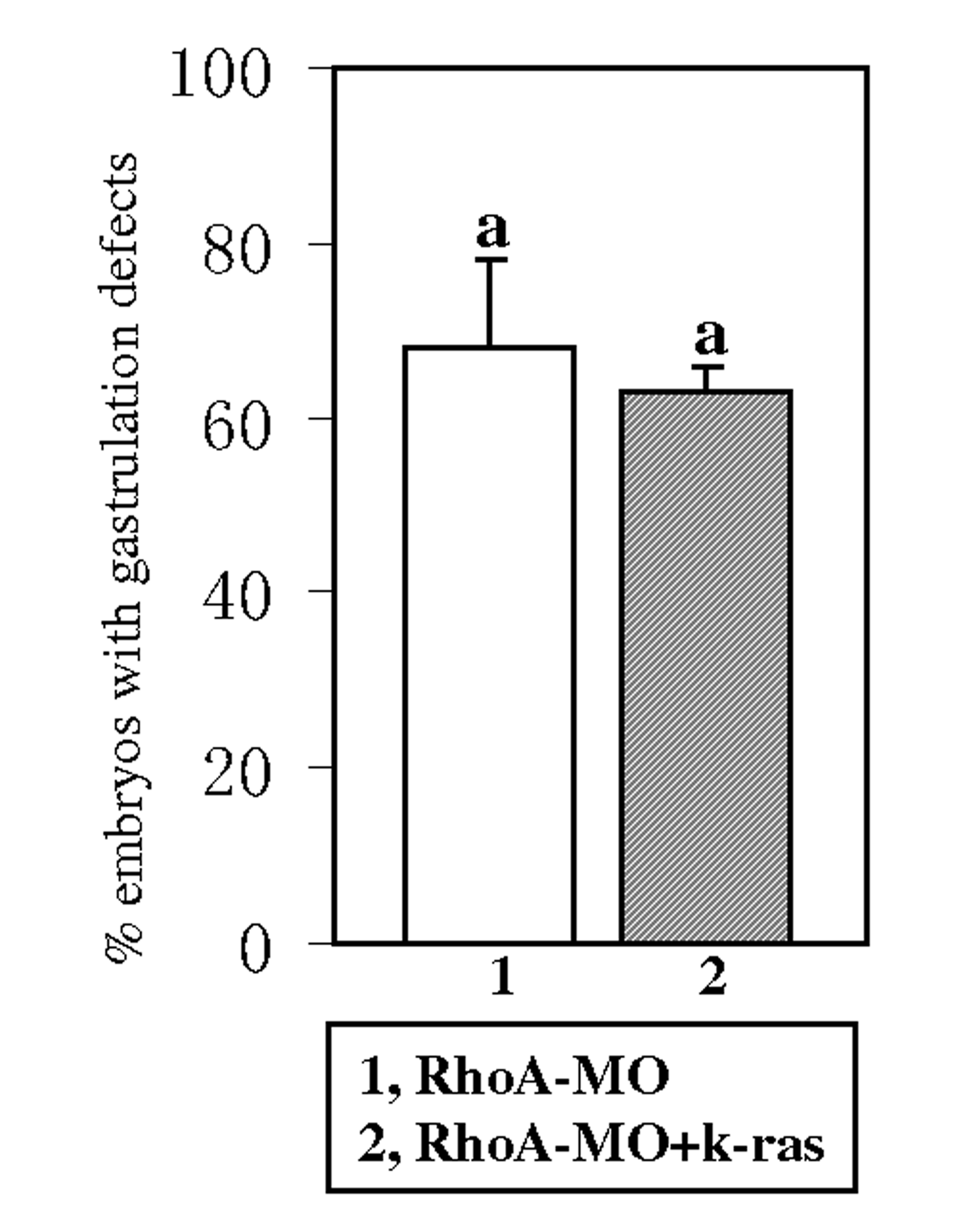

Supplement: Figure S7 — k-ras mRNA could not rescue the gastrulation defects induced by RhoA knock down. RhoA-MO injection can induce gastrulation defects [12] and these defects could not be rescued by the co-injection of k-ras mRNA. Embryos were observed at 1-somite stage. Embryo numbers n1 = 113, n2 = 112, from 2 sets of independent experiments. Data are means±SD. Values indicated by the same letter are not significantly different at p<0.05. (9.69 MB TIF) [file pone.0002850.s007.tif]

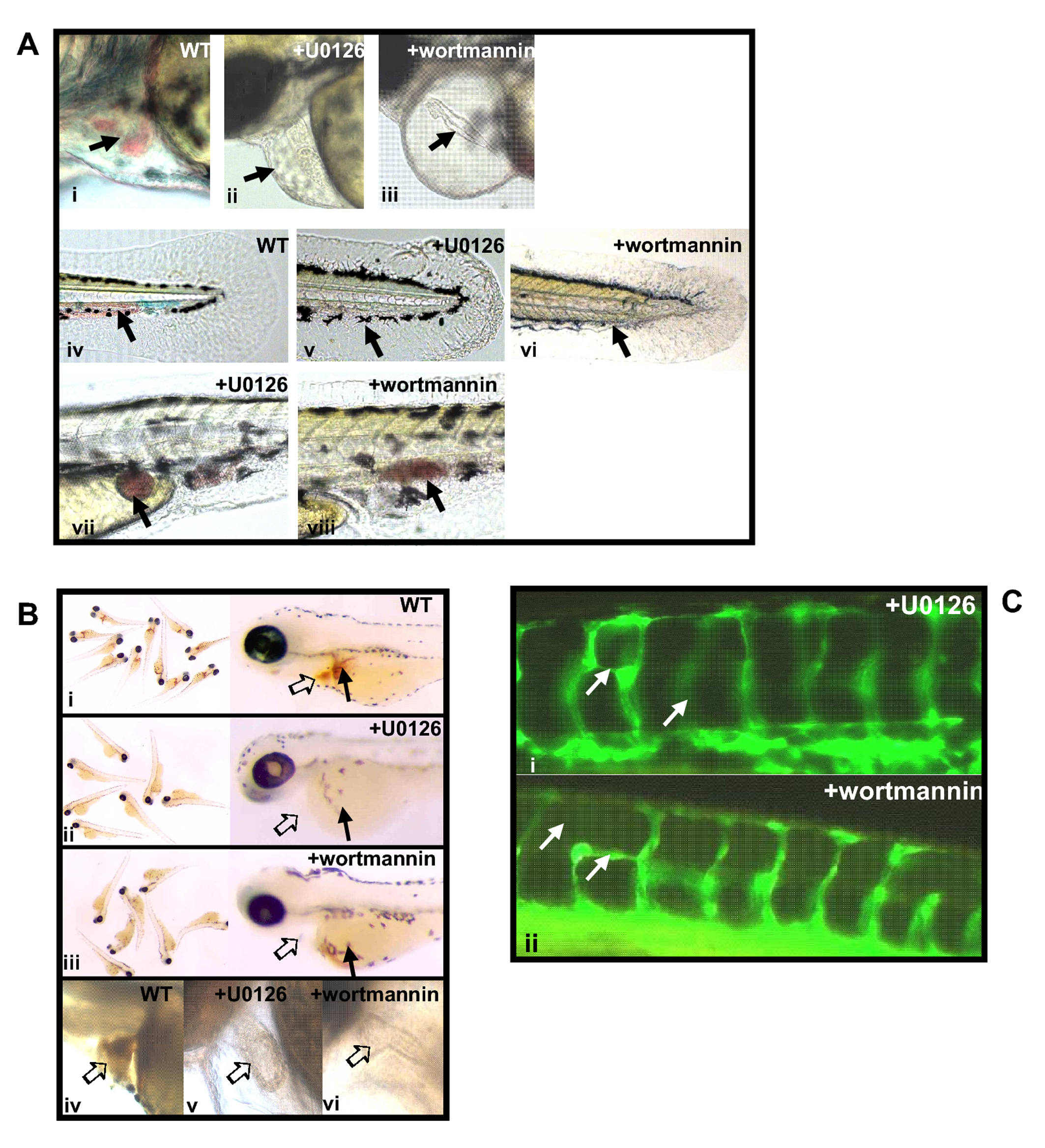

Supplement: Figure S8. — PI3K inhibitor wortmannin or MEK inhibitor U0126 could induce hematopoietic and angiogenic defects similar to the defects induced by K-ras knock-down. (A) Either wortmannin or U0126 treatment were able to cause the hematopoietic defects. These defects include empty heart, with no or few red blood cells inside heart (indicated by arrows in ii and iii, compared to wild type in i), reduced or lack of normal circulation and reduced number of circulating red blood cells (indicated by arrows in v and vi, compared to wild type in iv), and accumulation of blood cells in some sites away from the circulation (indicated by arrows in vii and viii). All embryos were observed at 4 dpf (days-post fertilization), lateral view, anterior to the left and dorsal to the top. (B) o-Dianisidine staining for wortmannin or U0126 treated embryos, showing loss or reduction of hemoglobin positive cells overall, especially inside heart and in yolk sac (indicated by empty arrows and block arrows respectively in ii, iii, v and vi, compared to wild type embryos in i and iv). Except for grouped embryos, all other embryos are lateral view, anterior to the left and dorsal to the top. Embryos were observed at 6 dpf. (C) Either wortmannin or U0126 treatment were able to cause angiogenic defects. Inhibitor treatment for fli1-GFP embryos resulted in disorganized blood vessels, including the missing segmental vessels and/or bearing ectopic vessel sprouts (indicated by arrows in i and ii), similar to the defects caused by K-ras knock-down. Embryos were observed at 4 dpf, lateral view, anterior to the left and dorsal to the top. (5.06 MB TIF) [file pone.0002850.s008.tif]

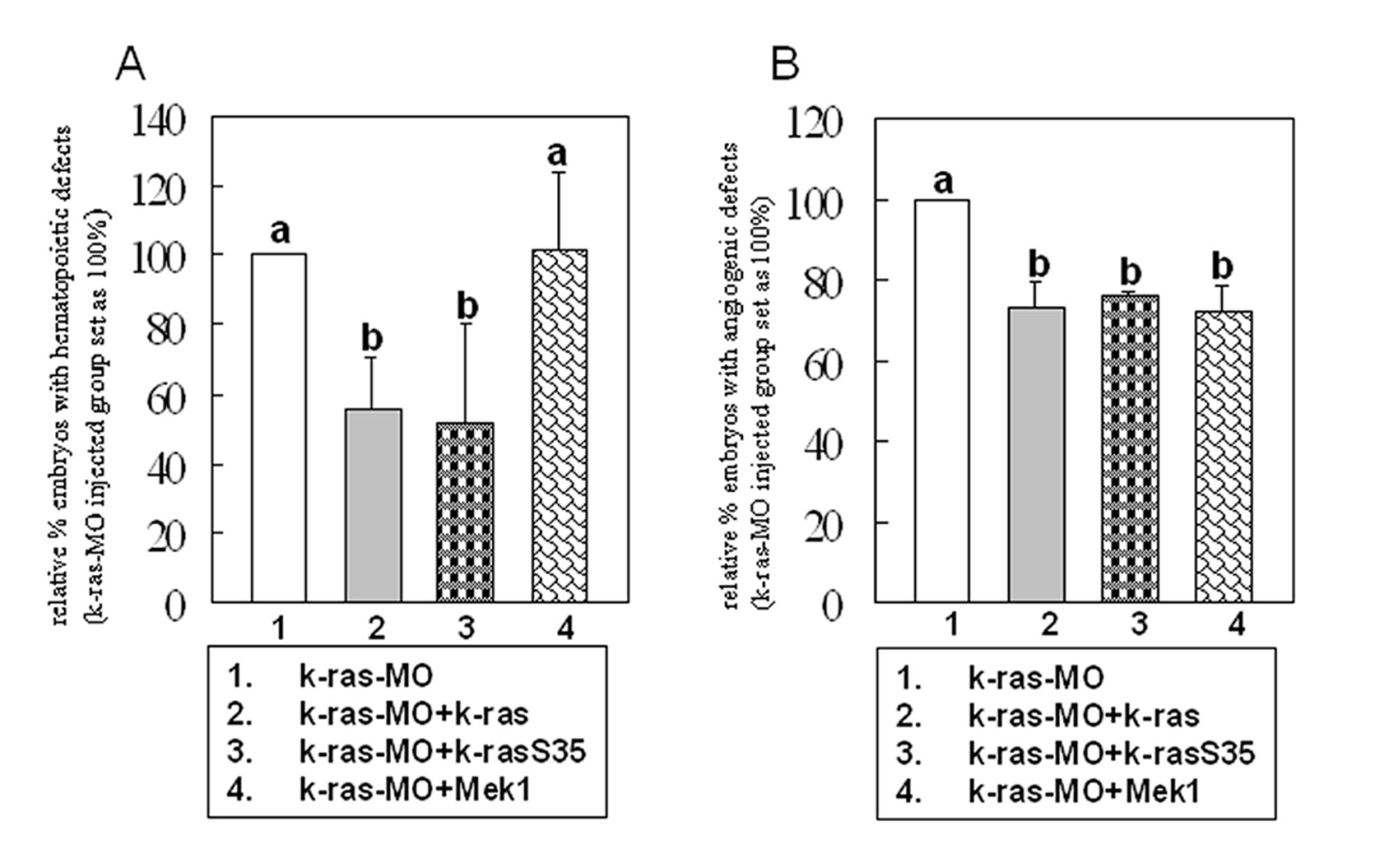

Supplement: Figure S9 — Raf is involved in mediating K-ras signaling for both hematopoiesis and angiogenesis, while Mek might be only involved in angiogenesis, but not in hematopoiesis. (A) Hematopoietic defects caused by K-ras knockdown could be rescued by wild type k-ras and k-ras mutant k-rasS35 respectively, but not Mek1. Embryo numbers n1>500, n2 = 475, n3 = 142 and n4 = 204, from >2 independent sets of experiments. (B) Angiogenic defects caused by K-ras knockdown could be rescued by wild type k-ras, k-ras mutant k-rasS35 and Mek1 respectively. Embryo numbers n1 = 133, n2 = 92, n3 = 39 and n4 = 89 from >2 independent sets of experiments. All data are means±SD (standard deviation). Values indicated by the same letter are not significantly different at p<0.05. (7.49 MB TIF) [file pone.0002850.s009.tif]
