# Supplementary material for: K-ras/PI3K-Akt Signaling Is Essential for Zebrafish Hematopoiesis and Angiogenesis
Source: PLoS One. 2008 Aug 6;3(8):e2850. doi: 10.1371/journal.pone.0002850 (PMC2483249; doi:10.1371/journal.pone.0002850)
Supplement: Text S1 — (0.05 MB DOC) [file pone.0002850.s016.doc]

**Supporting information**

## Materials and methods

***k-ras* gene isolation.** Since the N-terminal is highly conserved among Ras protein members, we used known zebrafish *N-ras* as query sequence to search NCBI est (Expressed Sequence Tags) databases. From the blasting results, we found that [AW421285](http://www.ncbi.nlm.nih.gov/entrez/viewer.fcgi?db=nucleotide&val=6949217) (fj89d12.y1) is likely a potential candidate of K-RAS homologue. Based on the sequence of fj89d12.y1 and fj89d12.x1, we designed two primers 5’CGTTGATATT TTGGGGTGAAG and 5'GGACCAACGA CACAACAGTG, and then performed PCR using 20hpf (hours-post fertilization) embryo cDNA as template. One PCR product above 1kb was obtained and subsequent analysis confirmed that this 1kb cDNA covers the full coding region of zebrafish *k-ras* gene.

Alignment and phylogenetic analysis. Phylogenetic tree generation and sequence alignment were performed using ClustalW program. Genbank accession numbers for these sequences are zebrafish K-ras DQ486868, zebrafish N-ras U62619, zebrafish BC048875, human K-RAS-2A P01116, human K-RAS-2B P01118, human H-RAS P01112, human N-RAS P01111, mouse K-RAS2 NP_067259, mouse H-RAS NP_032310 and mouse N-RAS NP_035067 respectively.

Whole-mount *in* *situ* hybridization. The fragments covering 5’UTR plus partial coding region of *k-ras* were obtained through RT-PCR and then were cloned into pGMET-easy vector for generating antisense and sense RNA probes for whole mount *in situ* hybridization. Digoxigenin-labled antisense RNA probes were synthesized based on the manufacturer’s instruction (Roche). Whole mount *in-situ* hybridization assay was performed as described (28).

RT-PCR analysis.

RT-PCR was performed to analyze the expressions of three zebrafish *ras* isoforms. Fragments covering the partial 5’UTR plus partial coding region were amplified using the primers based on their corresponding UniGene sequences (UniGene Dr.20193 for *k-ras*, UniGene Dr.233 for *n-ras* and UniGene Dr.20193, for BC048875 respectively). These primers are 5’GCCGGTGATCGCATAGTATCG and 5’ACGTTTCTCCGTCAATCACC for *k-ras*; 5’GGATTGTTAACGCGCTCTTC and 5’GCCAGACTTGTTTAACGGCTG for *n-ras;* and 5’CTTAGCCGAGAGGAGCTAATGC and 5’AGTGCACTTTTCCCAACACC for BC048875 respectively. ß-actin transcripts were amplified in the same reaction as a control using primers 5’TCCTATGTGGGAGATGAGGC and 5’CTTCTGCATACGGTCAGCAA. Total RNA was extracted from staged embryos and different tissues of adult using the TRIzol Reagent (Life Technologies, USA) according to the manufacturer’s instruction. cDNA was synthesized from the total RNA using AMV reverse transcriptase (Promega). PCR was carried out following the program 94ºC, 30”; 60ºC, 30” and 68ºC, 30” with 25 cycles. Negative controls were performed in parallel, except for running 5 more additional cycles, for each of them by applying RNA as templates for PCR directly, intending to rule out the probable contamination of genomic DNA.

**Morpholino sequence**. k-ras-MO1 sequence is 5’-CATTCTTCACCCCAAAATATCAACG, and its mismatch control MO1-mis sequence is 5’-CATTGTTCAGCCCATAATAACAACG. k-ras-MO2 sequence is 5’- CGGCTATTTTCAGTAGGTGGAGGCG. RhoA-MO sequence is 5’- TCCGTCGCCTCTCTTATGTCCGATA.

**mRNA and plasmid constructs for injection.** The open reading frame of zebrafish wild type *k-ras*, *n-ras* were cloned into pcDNA3.1(+) (Invitrogen). Mutation constructs, k-rasN17, k-rasC40 and k-rasS35 were generated using QuikChange® Site-Directed Mutagenesis Kit (Stratagene) by following manufacturer’s instruction and using k-ras-pcDNA3.1(+) as template. Open reading frame of zebrafish *akt2* (Genbank AY056465) was cloned into pGEMT-Easy (Promega). mRNA of each of the above was generated using mMESSAGE mMACHINE SP6/T7 transcription kit and Poly (A) tailing kit (Ambion) respectively. Rat *Mek1* cDNA was cloned into pXJ40 vector and was injected directly. k-rasN17 plasmid was also injected directly.

**Western Blot Analysis.** **Western** blots analysis were carried out by using human ß-actin antibody (1:2000, Sigma), K-ras antibody, H-ras antibody and N-ras antibody respectively (1:500, Santa Cruz Biotechnology).

**Microscopy and image analysis.** DIC and fluorescence microscopy were performed on a Zeiss Axiovert 200M inverted microscope and images were acquired by using AxioVision 4.0 software. Adobe Photoshop 7.0 was used to process all images.

# Legends for supporting figures and movies

**Figure S1. Comparison of zebrafish *k-ras*, *n-ras* and BC048875.**

(A) Comparison of amino acid sequences of three zebrafish Ras proteins, K-ras, N-ras and BC048875.

(B) Alignment of 5’UTRs of three zebrafish *ras* isoforms, *k-ras*, *n-ras* and BC048875. The morpholino targeting sites specific for *k-ras, n-ras* and BC048875 were highlighted with shade.

**Figure S2. Expression analyses of zebrafish *k-ras* in tissues*.***

RT-PCR analysis of zebrafish *k-ras, n-ras* and BC048875 expression in adult zebrafish tissues. Most tissues examined, except spleen, show high or medium level of *k-ras* expression. Zebrafish *n-ras* and zebrafish BC048875 transcripts were also detectable in all tissues examined at variant levels.

**Figure S3. Reduced heart beat rate was induced by K-ras knock-down, and it was able to be rescued by *k-ras* mRNA co-injection.**

The observed heart beat rate (per 30 seconds) at 30hpf (hours-post fertilization) of wild type embryos, k-ras-MO injected embryos, and k-ras-MO plus k-ras mRNA co-injected embryos respectively, showing the reduced heart beat rate caused by K-ras knock-down and the rescue by k-ras mRNA co-injection. Embryo numbers, n1=30, n2=26 and n3=38. Data are means ± SD (standard deviation), *p<0.05.

Values indicated by the same letter are not significantly different at p<0.05.

**Figure S4. The injection of mis-match k-ras morpholino (k-ras-MO-mis) could not induce the defects caused by k-ras morpholino.**

(A) K-ras-MO-mis injected embryos showed significant difference from K-ras-MO injected embryos by the analysis of hematopoietic defects. Embryo numbers n1=106, n2=279 and n3>500 from >2 sets of independent experiments.

(B) K-ras-MO-mis injected embryos showed significant different from K-ras-MO injected embryos by the analysis of angiogenic defects. Embryo numbers n1=81, n2=117 and n3=113 from >2 sets of independent experiments.

Data are means ± SD. Values indicated by the same letter are not significantly different at p<0.05.

**Figure S5. Determination of K-ras, N-ras and H-ras protein level between wild type and k-ras-MO injected embryos.**

K-ras-MO injected embryos (1dpf, one day post fertilization) showed reduced K-ras protein expression compared to its wild type control, while the expression of N-ras and H-ras was not affected significantly, indicating the specificity and efficiency of K-ras knock-down.

**Figure S6. RFP expression analysis at 20hpf for k-ras-5’UTR-RFP injected embryos, indicating the targeting specificity of *k-ras* morpholino antisense oligo.**

(A) Embryo injected with k-ras-5’UTR-RFP/PCS (red fluorescent protein reporter was down stream of K-ras 5’UTR and was cloned into PCS2 vector) construct, showing strong RFP signal.

(B) Embryo co-injected with k-ras-5’UTR-RFP/PCS and k-ras-MO1, showing very weak RFP signal, indicating the blockage of RFP protein expression by k-ras-MO1.

(C) Embryos from different treatments, showing the different RFP strength under the same exposure. These embryos were (i), injected with k-ras-5’UTR-RFP/PCS alone; (ii), co-injected with k-ras-5’UTR-RFP/PCS and k-ras-MO1; and (iii), wild type embryo with no injection.

**Figure S7. k-ras mRNA could not rescue the gastrulation defects induced by RhoA knock down.**

RhoA-MO injection can induce gastrulation defects (12) and these defects could not be rescued by the co-injection of k-ras mRNA. Embryos were observed at 1-somite stage. Embryo numbers n1=113, n2=112, from 2 sets of independent experiments. Data are means ± SD. Values indicated by the same letter are not significantly different at p<0.05.

**Figure S8. PI3K inhibitor wortmannin or MEK inhibitor U0126 could induce hematopoietic and angiogenic defects similar to the defects induced by K-ras knock-down.**

(A) Either wortmannin or U0126 treatment were able to cause the hematopoietic defects. These defects include empty heart, with no or few red blood cells inside heart (indicated by arrows in ii and iii, compared to wild type in i), reduced or lack of normal circulation and reduced number of circulating red blood cells (indicated by arrows in v and vi, compared to wild type in iv), and accumulation of blood cells in some sites away from the circulation (indicated by arrows in vii and viii). All embryos were observed at 4dpf (days-post fertilization), lateral view, anterior to the left and dorsal to the top.

(B) o-Dianisidine staining for wortmannin or U0126 treated embryos, showing loss or reduction of hemoglobin positive cells overall, especially inside heart and in yolk sac (indicated by empty arrows and block arrows respectively in ii, iii, v and vi, compared to wild type embryos in i and iv). Except for grouped embryos, all other embryos are lateral view, anterior to the left and dorsal to the top. Embryos were observed at 6dpf.

(C) Either wortmannin or U0126 treatment were able to cause angiogenic defects. Inhibitor treatment for fli1-GFP embryos resulted in disorganized blood vessels, including the missing segmental vessels and/or bearing ectopic vessel sprouts (indicated by arrows in i and ii), similar to the defects caused by K-ras knock-down. Embryos were observed at 4dpf, lateral view, anterior to the left and dorsal to the top.

**Figure S9. Raf is involved in mediating K-ras signaling for both hematopoiesis and angiogenesis, while Mek might be only involved in angiogenesis, but not in hematopoiesis.**

(A) Hematopoietic defects caused by K-ras knockdown could be rescued by wild type *k-ras* and *k-ras* mutant k-rasS35 respectively, but not Mek1. Embryo numbers n1>500, n2=475, n3=142 and n4=204, from >2 independent sets of experiments.

(B) Angiogenic defects caused by K-ras knockdown could be rescued by wild type *k-ras*, *k-ras* mutant k-rasS35 and Mek1 respectively. Embryo numbers n1=133, n2=92, n3=39 and n4=89 from >2 independent sets of experiments.

All data are means ± SD (standard deviation). Values indicated by the same letter are not significantly different at p<0.05.

**Legends for supporting movies:**

**K-ras knock down resulted in reduced circulation of blood cells, and reduced heart beating rate**

**Movie S1.** Beating heart filled with plenty of red blood cells in wild type embryo. Embryo at 2dpf (days-post fertilization).

**Movie S2.** Beating heart filled with few red blood cells in k-ras-MO injected embryo. Embryo at 2dpf.

**Movie S3**. Plenty of red blood cells circulating inside dorsal aorta and posterior cardinal vein in wild type embryo. Embryo at 2dpf.

**Movie S4.** Few red blood cells circulating inside dorsal aorta and posterior cardinal vein in k-ras-MO injected embryo. Embryo at 2dpf.

**Movie S5**. Plenty of red blood cells circulating inside caudal artery and caudal vein in wild type embryo. Embryo at 2dpf.

**Movie S6.** Few red blood cells circulating inside caudal artery and caudal vein in k-ras-MO injected embryo. Embryo at 2dpf.
