# Supplementary material for: K-ras/PI3K-Akt Signaling Is Essential for Zebrafish Hematopoiesis and Angiogenesis
Source: PLoS One. 2008 Aug 6;3(8):e2850. doi: 10.1371/journal.pone.0002850 (PMC2483249; doi:10.1371/journal.pone.0002850)
Supplement: Table S1 — Statistic results summarizing similar hematopoietic defects induced by k-ras-MO1, k-ras-MO2 and k-ras-N17 respectively, indicating that the hematopoietic defects are closely related to the disruption of K-ras signaling. (0.03 MB DOC) [file pone.0002850.s017.doc]

**Table S1.**  Statistic results summarizing similar hematopoietic defects induced by k-ras-MO1, k-ras-MO2 and k-ras-N17 respectively, indicating that the hematopoietic defects are closely related to the disruption of K-ras signaling.
