# Supplementary material for: K-ras/PI3K-Akt Signaling Is Essential for Zebrafish Hematopoiesis and Angiogenesis
Source: PLoS One. 2008 Aug 6;3(8):e2850. doi: 10.1371/journal.pone.0002850 (PMC2483249; doi:10.1371/journal.pone.0002850)
Supplement: Table S2 — Hematopoietic defects induced by K-ras knock-down could be partially rescued by over-expression of k-ras mRNA, suggesting the specificity of K-ras knock-down. (0.03 MB DOC) [file pone.0002850.s018.doc]

**Table S2.**  Hematopoietic defects induced by K-ras knock-down could be partially rescued by over-expression of *k-ras* mRNA, suggesting the specificity of K-ras knock-down.
