# Supplementary material for: K-ras/PI3K-Akt Signaling Is Essential for Zebrafish Hematopoiesis and Angiogenesis
Source: PLoS One. 2008 Aug 6;3(8):e2850. doi: 10.1371/journal.pone.0002850 (PMC2483249; doi:10.1371/journal.pone.0002850)
Supplement: Table S3 — Treatments with wortmannin or U0126 for zebrafish embryos could induce the hematopoietic defects and angiogenic defects, which phenocopy the functional loss of K-ras. (0.03 MB DOC) [file pone.0002850.s019.doc]

**Table S3, PI3K inhibitor wortmannin and MEK inhibitor U0126 treatments can phenocopy the functional loss of K-ras.**

| **treatments** | **phenotype** | | |
| --- | --- | --- | --- |
|  | Live embryos at 2.5dpf | O-D staining embryos at 3dpf | fli-1 transgenic line  2.5 dpf |
| U0126(10μM), started from 3hpf | No or very faint red color visible inside beating heart, poor circulation through the body (n=35) | No staining in the heart and no or severely reduced staining for the circulation blood cells in the yolk (n=10) | Abnormal branching of segmental vessels with ectopic branch sprouts and/or with missing vessels (n=33) |
| Wortmannin(1μM), started from 3hpf | No or very faint red color visible inside beating heart, poor circulation through the body (n=46) | No staining in the heart and severely reduced staining for the circulation blood cells in the yolk  (n=8) | Abnormal branching of segmental vessels with ectopic branch sprouts and/or with missing vessels (n=25)  *wortmannin concentration for this treatment is 500nM |

**Table S3**. treatments with wortmannin or U0126 for zebrafish embryos could induce the hematopoietic defects and angiogenic defects, which phenocopy the functional loss of K-ras.
